# Increasing Outreach and Expanding Inclusion: Community-Based Educational Gerontology

**DOI:** 10.1093/geroni/igaa057.1755

**Published:** 2020-12-16

**Authors:** Jennifer Sasser

**Affiliations:** Human Development & Family Sciences, Oregon State University, Portland, Oregon, United States

## Abstract

Gerontologists have the opportunity to step into an increasingly significant role as public educators who convene gatherings focused on expanding aging awareness and literacy, inter-generational inquiry and collaboration, and age inclusion, equity and justice. The purpose of this presentation is to share creative design principles and keen take-aways from several ongoing community-based educational interventions connected to these themes. As well, we will discuss the role such public-facing initiatives might play in making a compelling case for the importance of supporting and participating in various kinds of formal educational pathways in the field of aging. Part of a symposium sponsored by the Community College Interest Group.

